# Regulation of hippocampal progenitor cell survival, proliferation and dendritic development by BDNF

**DOI:** 10.1186/1750-1326-4-52

**Published:** 2009-12-21

**Authors:** Se Hoon Choi, Yun Li, Luis F Parada, Sangram S Sisodia

**Affiliations:** 1 Committee on Neurobiology, University of Chicago, Chicago, Illinois 60637, USA; 2 Department of Neurobiology, University of Chicago, Chicago, Illinois 60637, USA; 3 Department of Developmental Biology & Kent Waldrep Center for Basic Research on Nerve Growth and Regeneration, University of Texas Southwestern Medical Center, Dallas, Texas 75390, USA; 4 Current address: The Whitehead Institute for Biomedical Research, Cambridge, Massachusetts 02142, USA

## Abstract

**Background:**

Environmental enrichment (EE) is known to enhance BDNF levels and neurogenesis in the adult hippocampus. To examine the role of BDNF in modulating EE-mediated adult hippocampal neurogenesis, we conditionally ablated *BDNF *expression in the hippocampus (cKO mice) and have assessed proliferation, survival, differentiation and dendritic development of hippocampal progenitors.

**Results:**

We show that while the extent of cell proliferation and neuronal fate differentiation in the hippocampus of cKO mice is not different from wild-type (WT) littermates maintained in either standard or enriched conditions, reduced BDNF levels significantly impaired the survival of newborn cells in both housing conditions. In addition, while highly active enriched WT mice exhibited a robust increase in progenitor cell proliferation, highly active cKO mice showed a modest increase in cell proliferation compared to standard housed or underactive cKO mice.

**Conclusions:**

There results argue that while BDNF plays a role in exercise-induced cell proliferation, other factors must contribute to this phenomenon. We also show that dendritic development was impaired in cKO mice maintained in standard housing conditions, and that EE rescued this phenotype.

## Background

Neurons are continuously added to the hippocampal dentate gyrus (DG) throughout life. Studies have correlated increased neurogenesis in the DG with improved performance on hippocampal learning tasks [1], suggesting that adult-generated neurons can positively impact adult hippocampal function. Adult hippocampal neurogenesis is a dynamic process that is regulated both positively and negatively, by a variety of growth factors and environmental experiences [2].

Brain-derived neurotrophic factor (BDNF) is highly expressed in hippocampus [3], but the functional role of this neurotrophin in the adult hippocampus, and specifically with respect to the proliferation and survival of neural precursor cells (NPCs) in the subgranular cell layer (SGL) of the DG, is controversial. For example, studies have shown that heterozygous *BDNF *knockout (*BDNF+/-*) mice exhibit reduced [4] or enhanced cell proliferation [5] in the DG. Similarly, basal levels of cell survival were shown to be impaired in two studies [4,5], but not in another [6]. However, the interpretation of these latter series of studies is confounded by the fact that *BDNF+/- *mice exhibit a multiplicity of growth, metabolic, neuronal maturation and behavioral abnormalities [7-10] and it is very plausible that these phenotypes could have a significant influence on adult neurogenesis.

It is now clear that exposure of adult rodents to environmental enrichment (EE) and exercise induces neurogenesis in the DG [11,12] and is correlated with an elevation in hippocampal BDNF levels [13,14]. To examine the influence of BDNF on maintaining basal levels of neurogenesis and/or EE-mediated neurogenesis, we examined mice in which BDNF expression was conditionally eliminated in mature neurons of the adult hippocampus (cKO). We found that in both standard housing and EE conditions, the proliferation and neuronal differentiation of hippocampal NPCs in cKO mice and wild-type (WT) littermates were indistinguishable. On the other hand, the survival of the NPCs was significantly impaired in cKO mice that were housed in either condition. Notably, while running wheel exercise clearly mediated enhanced NPC proliferation in WT mice, exercise-mediated NPC proliferation in cKO mice was elevated, but only to a moderate level. Furthermore, while dendritic development was altered in cKO mice housed in standard conditions, these impairments were rescued by EE. Thus, we conclude that BDNF plays a critical role in regulating the survival and dendritic development of NPCs in the adult hippocampus. More importantly, exercise-induced NPC proliferation is only modestly impacted by expression of BDNF, findings which would suggest that additional factors play a role in this process.

## Results

### Analysis of BDNF levels in hippocampus of WT and cKO mice

We previously demonstrated that conditional ablation of floxed *PSEN1*, encoding presenilin 1, by a CamKII-driven *Cre *(line T29-1, [15]) transgene is initiated postnatally, and results in complete loss of *PSEN1 *expression in mature neurons in the hippocampal formation by the age of 3 months [16]. Thus, we placed cohorts of 3-month-old male *BDNF*^2*lox*^/*BDNF*^2*lox*^/*CamKII-Cre *mice (cKO) and male *BDNF*^2*lox*^/*BDNF*^2*lox *^mice (WT) in EE for 1 month (referred as "enriched" mice) and maintained additional cohorts of cKO and WT mice in standard conditions for 4 months (referred as "standard" mice).

**Figure 1:**
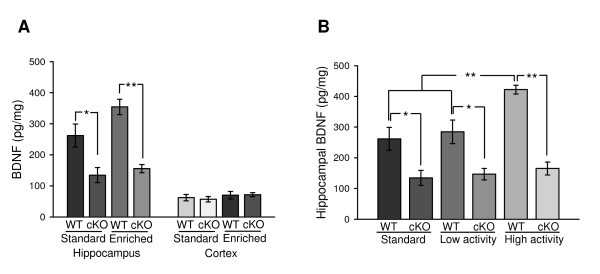
**cKO mice show reduced hippocampal BDNF levels**. ***A***, Quantification of BDNF protein levels (Mean ± SEM, pg/mg). 5-13 animals/group. *, *P *< 0.05; **, *P *< 0.01. ***B***, Levels of hippocampal BDNF after sub-grouping the EE groups into low-activity and high-activity groups. *, *P *< 0.05; **, *P *< 0.01.

We now show that standard housed cKO mice exhibit an approximately 50% reduction of hippocampal BDNF levels compared to standard WT mice (Fig. 1A). The residual levels of hippocampal BDNF in cKO mice might reflect expression in non-neuronal cells, in which the floxed *BDNF *alleles are not deleted by the neuronal-specific *CamKII-Cre *allele, or that this represents BDNF that is axonally transported to the hippocampus from cortical regions in the CNS [17] and further studies will be required to distinguish between these, or other, models. In any event, and as shown earlier [14], BDNF is elevated in the cohort of enriched WT mice, albeit to levels that did not reach statistical significance (Fig. 1A). On the other hand, BDNF was not elevated in the hippocampus of enriched cKO mice. As a control, we assessed BDNF levels in cortical tissue and failed to observe any differences in BDNF levels either within, or between groups (cortex; Fig. 1A).

It has been shown that hippocampal BDNF levels are increased upon long-term (1 year; [14]) but not short-term (2 month; [18]) EE. On the other hand, 5-day [13] or 7-day [19] running wheel exercise was sufficient to increase hippocampal BDNF levels, suggesting that running might be a critical factor in the EE paradigm that is required for inducing hippocampal BDNF levels. Hence, we assessed the impact of running wheel activity on BDNF levels in our enriched mice. Notably, the activity levels, defined as the percent time spent on the running wheels, in cKO mice (26.51 ± 1.67%) were not different from those of WT mice (26.78 ± 1.75%). We chose an arbitrary cutoff of 25% average activity because the BDNF levels in enriched mice that showed activities lower than this value were essentially identical to those of standard mice in both the WT and cKO groups (Fig. 1B). We divided the enriched mice into "high-activity", defined as activity greater than 25%, and "low-activity" groups, defined as activity less than 25%. It should be noted that mice exhibiting between 23.4% and 29.3% activity showed highly variable results with respect to BDNF levels and this group was not included for further analysis. Whereas we observed no differences in BDNF levels between standard WT mice and low-activity WT mice, high-activity WT mice showed a significant increase in BDNF levels compared to standard WT mice and low-activity WT mice (Fig. 1B). Thus, while our relatively short 30-day EE paradigm did not result in significant elevations in hippocampal BDNF in the total cohort of mice (Fig. 1A), "highly-active" WT mice clearly exhibit marked increases in hippocampal BDNF levels. As expected, the levels of hippocampal BDNF levels in standard cKO, low-activity cKO and high-activity cKO mice cohorts were essentially indistinguishable.

### Behavioral Analysis of cKO and WT mice

**Figure 2:**
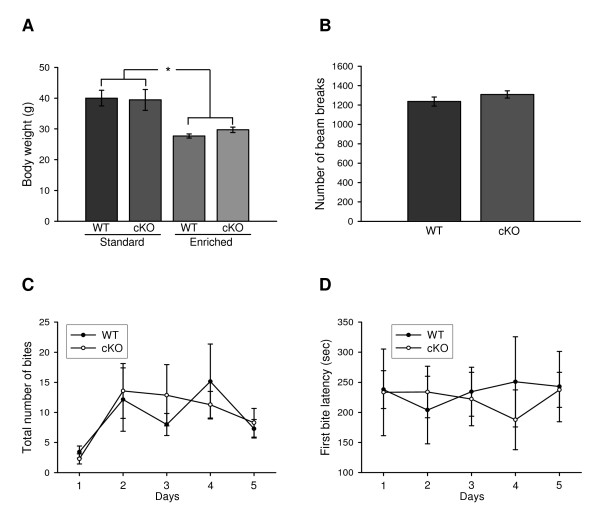
**cKO mice are not obese, anxiety-prone nor hyperactive**. cKO mice are not obese, anxiety-prone nor hyperactive. ***A***, Body weights in WT and cKO mice. 7-13 animals/group. *, *P *< 0.05. ***B***, Locomotor activity in WT and cKO mice. 7 animals/group. ***C-D***, Total number of bites (***C***) and first bite latency (***D***) in the resident intruder paradigm. 7 animals/group.

In view of the reports documenting that *BDNF+/- *mice exhibit growth retardation, obesity, hyperactivity and aggressiveness [7-10], we felt it was critical to assess these parameters in our cKO mice that accumulate hippocampal BDNF to 50% of the levels observed in WT animals. We failed to observe any differences in body weights (Fig. 2A), locomotor activity in open field (Fig. 2B) and levels of aggression in resident intruder tests (Fig. 2C and 2D) between 4-month-old cKO and WT strains.

### Reduced BDNF levels does not impair basal and EE-induced NPC proliferation

In order to examine the impact of BDNF on NPC proliferation, cohorts of 3-month-old male cKO or WT mice were subject to EE for 1 month, after which time, mice were injected with BrdU and sacrificed after 1 day. Parallel cohorts of mice were sacrificed 4 weeks after BrdU injection in order to determine the survival rate and differentiation of newborn NPCs.

**Figure 3:**
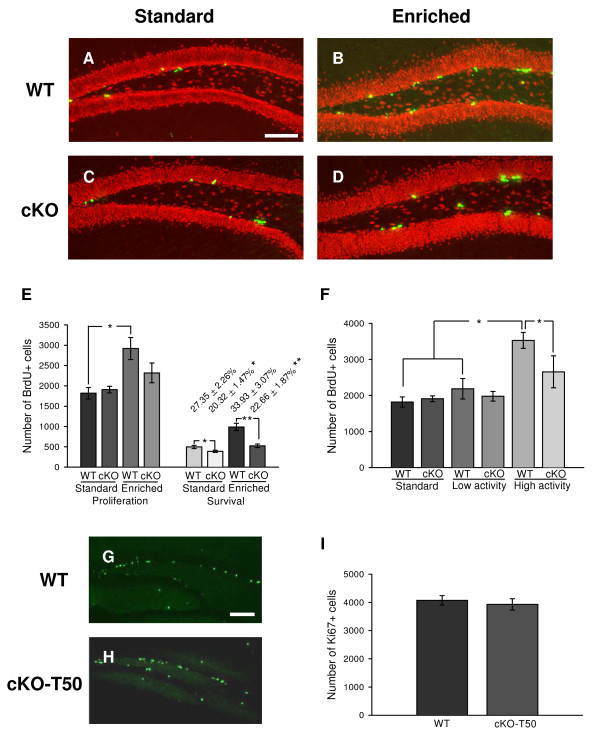
**Reduced BDNF levels affect cell survival and exercise-mediated cell proliferation**. ***A*-*D***, Photomicrographs of BrdU^+ ^cells 1 day after BrdU injection in the DG of WT (***A ***and ***B***) and cKO (***C ***and ***D***). The mice were maintained in either standard (***A ***and ***C***) or enriched conditions (***B ***and ***D***). Green or yellow, BrdU^+ ^cells; Red, NeuN^+ ^cells. Scale bar: 100 μm. ***E***, Quantification of BrdU^+ ^cells (Mean ± SEM; 6-12 animals/group). *, *P *< 0.05; **, *P *< 0.01. ***F***, Quantification of BrdU^+ ^cells after sub-grouping the EE groups into low-activity and high-activity groups. *, *P *< 0.05. ***G*-*H***, Photomicrographs of Ki67^+ ^cells in the DG of WT (***G***) and cKO-T50 (***H***) mice in standard condition. Scale bar: 100 μm. ***I***, Quantification of Ki67^+ ^cells in cKO-T50 and control littermates (Mean ± SEM; 8 animals/group).

Examination of the number of BrdU^+ ^cells in cohorts of WT or cKO mice housed in standard conditions failed to reveal any statistically significant differences between groups (Fig. 3A and 3C, respectively; quantified in Fig. 3E). To provide additional support for these observations, we sought to examine the impact of conditionally ablating BDNF using a different mouse line expressing CamKII-Cre (line T50). The CamKII transgene in line T50 is active postnatally and complete deletion of floxed *BDNF *alleles in mature neurons occurs by two months of age [20], again leading to a 50% reduction in hippocampal BDNF levels [20]. As shown above for the *cKO-T29-1 *mice, precursor proliferation between the *cKO-T50 *and WT groups were indistinguishable (Figure 3G and 3H; quantified in Fig. 3I).

On the other hand, EE significantly increased the number of BrdU^+ ^cells in WT mice, as expected, but enriched cKO mice showed only a modest, yet non-significant increase in BrdU^+ ^cells compared to standard housed counterparts (Fig. 3B and 3D, respectively; quantified in Fig. 3E). Notably, while the slight elevation in BrdU^+ ^cells in enriched cKO mice is statistically insignificant relative to the enriched WT cohort (Fig. 3E), the fact that the levels of BrdU^+ ^cells are elevated in the cKO mice would suggest that factor(s) other than BDNF might contribute to EE-induced cell proliferation.

### Reduced BDNF levels impairs exercise-induced NPC proliferation

It has become increasingly evident that the critical apparatus that impacts on EE-mediated elevations of NPC proliferation is the running wheel [12]. Hence, we reexamined our earlier dataset by assessing the impact of running wheel activity on numbers of BrdU^+^cells in each group. We observed no significant differences in BrdU^+^cells in standard WT and low-activity WT cohorts (Fig. 3F). However, we observed a significant difference in numbers of BrdU^+ ^cells between the high-activity WT mice and standard WT mice or low-activity WT mice. In contrast, while we did observe a modest ~20% elevation in the number of BrdU^+ ^cells in the hippocampus of high-activity cKO mice compared to standard cKO mice or low-activity cKO mice, these differences were insignificant (Fig. 3F). More importantly, we now see a highly significant statistical difference in BrdU^+ ^cells in the hippocampus of high-activity cKO mice compared to high-activity WT mice. Thus, running wheel activity is critical for promoting NPC proliferation in the hippocampus of WT mice, and BDNF is at least, in part, a factor that is critical for regulating this process.

### Reduced BDNF levels impairs the survival, but not differentiation of newborn cells

SGL progenitors that reach maturity are fated to become granule neurons or glial cells [2]. To examine the impact of reduced BDNF levels on the survival and/or fate of newborn cells, cohorts of standard or enriched WT and cKO mice were sacrificed 4 weeks after the BrdU injection (i.e. at the 5-month time point). In order to provide a measure of cell survival during the 4-week post BrdU time period, we expressed the number of BrdU^+ ^cells at the 4-week post BrdU time point as a percentage of the number present at the 1-day post-BrdU time point (numbers above the survival groups in the Fig. 3E). This analysis revealed that a significantly lower fraction of BrdU^+ ^cells survived in cKO mice compared to WT mice housed in either standard or enriched conditions. Indeed, we observed a statistically significant decrease in the absolute numbers of surviving BrdU^+ ^cells in cKO compared to WT mice in both housing conditions.

**Figure 4:**
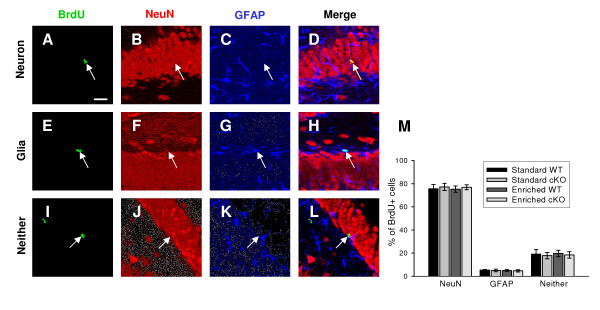
**Reduced BDNF levels do not affect neuronal fate differentiation**. ***A*-*L***, Confocal images of BrdU^+ ^cells in slices of WT or cKO mice. The slices were triply stained for the BrdU (***A***, ***E ***and ***I***), NeuN (***B***, ***F ***and ***J***), and GFAP (***C***, ***G ***and ***K***). The merged images of the three labels (***D***, ***H ***and ***L***) demonstrate cells with neuronal (***A***-***D***) or glial (***E***-***H***) properties or neither of them (***I***-***L***). Arrows indicate the position of BrdU^+ ^cells. Scale bar: 20 μm. ***M***, Percentage of BrdU^+ ^cells colabeled with NeuN or GFAP, or neither of them. 6-9 animals/group.

In order to determine the phenotypes of the BrdU^+ ^cells, we performed triple-labeling confocal immunohistochemical analysis using an antibody against BrdU, an antibody against the mature neuron-specific protein, NeuN, and an antibody against the astrocyte, GFAP (Fig. 4A - 4L). NeuN was consistently present in approximately 75% to 78% of BrdU^+ ^cells among all the groups, and no differences were observed in the fraction of BrdU^+ ^cells that were colabeled with NeuN within, or between groups (Fig. 4M). BrdU^+ ^cells that were colabeled with GFAP, or that were not co-labeled with either NeuN or GFAP showed no differences between these groups. Therefore, differentiation of progenitors to either neuronal or glial lineages appears not to be linked to the expression of BDNF and these effects are maintained whether the WT or cKO mice are maintained in standard or EE conditions. Finally, the fractional survival and neuronal differentiation of newborn cells in high-activity mice were not significantly different from those of low-activity mice in either the WT and cKO groups (data not shown).

### Dendritic development is impaired in standard cKO, but not in enriched cKO mice

Adult-generated neurons must develop dendrites to form connections with existing neurons to receive and convey information in order to integrate into the existing neural networks or rebuild neural circuitry. Previous studies have shown marked effects of BDNF on dendrite formation, but the results have not been conclusive. For example, while dendritic complexity in the hippocampal DG is increased in BDNF-overexpressing transgenic mice [21], BDNF has also shown to destabilize cortical dendrites [22]. BDNF can both induce and inhibit dendritic development depending on neuronal cell type and brain region [23,24]. Interestingly, EE has been shown to increase dendritic branching and length [25]. To examine whether BDNF is responsible for dendrite morphology in the standard condition and/or EE-mediated dendrite morphology, brain sections were immunostained with anti-DCX antibody, and dendritic length and complexity were analyzed.

**Figure 5:**
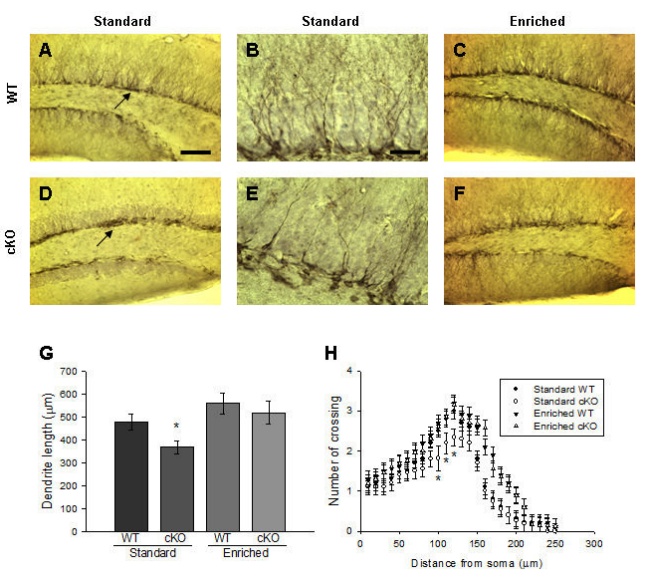
**Dendritic development is impaired in cKO mice in standard condition, but not in enriched condition**. ***A*-*F***, Photomicrographs of DCX^+ ^cells in the DG of WT (***A***, ***B ***and ***C***) and cKO (***D***, ***E ***and ***F***). The mice were maintained in either standard conditions (***A***, ***B***, ***D ***and ***E***) or enriched conditions (***C ***and ***F***). Scale bar: 80 μm in ***A ***or 20 μm in ***B***. ***G***-***H***, Quantification of total dendritic length (***G***) and dendritic complexity (***H***) of DCX^+ ^cells in WT and cKO mice (Mean ± SEM; 6 animals/group). *, *P *< 0.05.

While the majority of DCX^+ ^neurons in the DG of standard WT mice exhibited dendrites projecting to the granule cell layer or crossed the granule cell layer to the molecular layer, with the axis of the cell body perpendicular to the SGL (Arrow in Fig. 5A), a fraction of DCX^+ ^cell bodies in the standard cKO mice were located adjacent to SGL with the axis of the cell body parallel to the SGL (Arrow in Fig. 5D). DCX^+ ^immature neurons in standard cKO mice also displayed a significant decrease in total dendritic length compared to WT counterparts (Fig. 5G left two bars) and exhibited less complexity at 100-130 μm from the soma (Fig. 5B and 5E; quantified in Fig. 5H). However, after 8 weeks of enrichment (i.e, at the 5-month time point), these dendritic abnormalities in cKO mice were rescued (Fig. 5C and 5F; quantified in Fig. 5G right two bars), suggesting that while BDNF signalling is required for dendritic development in adult hippocampus in standard housing conditions, factor(s) in addition to BDNF impact upon EE-induced dendritic development. With respect to dendritic length, the EE paradigm showed a trend to increase the total length in WT mice, but these changes did not reach statistical significance (Fig. 5G). In addition, DCX^+ ^neurons in the enriched mice (both WT and cKO) were more complex than those in standard housed mice at 150-200 μm from the soma (Fig. 5H). Dendritic morphologies of high-activity mice did not show differences between low-activity mice in both WT and cKO groups.

## Discussion

Increasing evidence has provided support for the view that exposure of adult rodents to EE and exercise induces neurogenesis in the DG and elevates hippocampal BDNF levels. While several laboratories have tested the functional relevance of the correlation between EE and exercise-mediated neurogenesis and running, the emergent data has been largely contradictory and thus, inconclusive. In order to clarify the issue, we examined mice in which BDNF expression was conditionally eliminated in mature neuronal populations of the adult hippocampus and now offer several important insights.

First, we report that a 50% reduction in hippocampal BDNF levels following selective gene ablation in hippocampal neurons does not have a significant influence on cell proliferation in cohorts of animals housed in either standard or EE conditions. On the other hand, cell proliferation, mediated by high levels of physical exercise (running) is reduced in cKO mice compared to WT counterparts. Second, reduced BDNF level in cKO mice impairs both basal and EE-mediated survival of newborn cells, but does not affect neuronal fate differentiation in either housing condition. Third, dendritic development is impaired in cKO mice maintained in standard housing conditions, a phenotype that is rescued by EE. Taken together, our data suggest that BDNF expression in the adult hippocampus is required for precursor cell survival, exercise-induced cell proliferation, and dendritic development.

While hippocampal progenitor cell proliferation was not affected by reduced BDNF levels in the entire cohort of mice housed in either standard or EE conditions, we observe that exercise-induced cell proliferation was markedly impaired in cKO mice. While it has been reported that physical exercise up-regulates *BDNF *mRNA and encoded polypeptides in the hippocampus [13,26-28] and also induces cell proliferation [12], the molecular mechanism(s) that regulate exercise-mediated induction of BDNF expression and the cellular pathway(s) by which BDNF mediates cell proliferation are just beginning to emerge. For example, BDNF is an activity-induced gene that is transcriptionally regulated, and these transcriptional changes are initiated by Ca^2+ ^increases generated through the activation of NMDA receptors or voltage-sensitive Ca^2+ ^channels [29,30]. In this regard, it has been suggested that running wheel exercise may activate NMDA receptors in the hippocampus, and that in turn may enhance BDNF production. Supporting this notion, the enhancement of BDNF levels induced by running wheel exercise is suppressed in mice lacking the NMDA receptor β1 [31]. Running induces a specific increase in LTP in the DG that correlates with elevated cell numbers in the structure in adulthood [32]. BDNF plays a role in a form of LTP by enhancing glutamatergic synaptic transmission at presynaptic sites [33,34]. It should be noted that vascular endothelial growth factor (VEGF) and insulin growth factor-1 (IGF-1) are known to mediate the effects of exercise on neurogenesis and these effects appear primarily to increase cell proliferation [35]. Data from songbird indicate that VEGF acts upstream of BDNF to regulate adult neurogenesis [36] and peripheral administration of IGF-1 induces BDNF mRNA in rat brain [37]. Taken together, activation of BDNF-LTP, VEGF-BDNF and/or IGF-1-BDNF cascades may modulate cell proliferation.

While not established, it is likely that BDNF influences exercise-induced cell proliferation through TrkB that is highly expressed in the hippocampus [38]; the level of activation of this receptor is highly correlated with the availability of BDNF [39]. Moreover, the levels of full-length TrkB and phosphorylated TrkB in hippocampus have been shown to be increased by running exercise [40,41]. We have reported that *TrkB *mRNA and encoded polypeptides are expressed in neural progenitor cells and immature neurons in the adult hippocampal neurogenic niche, and that conditional ablation of TrkB from hippocampal neural progenitors using the *hGFAP-Cre *is sufficient to block running-induced increases in proliferation and neurogenesis [38]. These observations are consistent with the lack of proliferative response to running in the BDNF cKOs reported herein, and thus, we would argue that TrkB likely provides the signalling gateway through which BDNF induces proliferation upon high-level running activity.

Our finding that a 50% reduction of BDNF in our cKO mice impairs both basal and EE-mediated survival of newborn cells may be related to the known effects of BDNF on activation of phosphatidylinositide 3 kinase (PI3)/AkT (protein kinase B) kinase [42]. In this regard, AkT is activated by exercise [43], and inhibits the activities of proapoptotic proteins while enhancing the effects of a broad range of survival factors [44]. Future studies to delineate the relative contributions of AkT, or other signalling pathways, in BDNF-mediated survival of hippocampal progenitors are clearly warranted.

Quantification and comparison of overall dendritic development revealed that DCX^+ ^neurons in standard cKO mice exhibited decreases in total dendritic length compared to WT counterparts, similar to findings recently reported by Chan et al (2008) [45]. However, EE rescued the impaired dendritic development in cKO mice. Recently, Zhu et al (2009) have shown that BDNF+/- mice exhibited severe reduction in the dendritic spine density in the DG, and that this phenotype was also rescued by EE [46]. Together, these findings suggest that while BDNF signalling might play a critical role in dendritic development in standard housing conditions, synaptic changes due to EE can still occur in mice with low BDNF levels.

It is important to note that despite the strengths of our conclusions, studies by Chan et al (2008) have revealed that conditional inactivation of *BDNF *using a *CamKII-Cre *159 line [47] led to an increase in proliferation of newborn cells in the SGL in mixed cohorts of male and female mice, without any effects on cell survival in standard conditions [45]. In contrast to our findings in which we failed to observe any alterations in NPC proliferation by reducing BDNF levels in male mice, we note that Chan and colleagues reported an almost complete ablation of *BDNF *mRNA expression in the hippocampus, substantially lower than the 50% reductions in BDNF protein levels reported herein. We have previously shown that BDNF regulates eating behavior and locomotor activity [8], and that BDNF+/- mice that accumulate approximately half the levels of the normal gene product throughout their body indeed develop aggressiveness, hyperactivity and obesity [9,48]. The availability of conditional *BDNF *knockout mice has made it possible to assess how these behaviors are influenced by acute reductions in BDNF levels, in different brain areas and at different developmental stages. However, characterization of *BDNF *conditional knockouts has yielded mixed behavioral results. For example, the mice used by Chan and colleagues exhibit hyperactivity, obesity and aggressiveness [49], similar to the phenotypes seen in *BDNF*+/- mice. In contrast, we failed to observe any perturbations in these parameters in male mice of our strain (cKO-T29-1). Our cKO-T50 mice showed gender differences in depression-related behaviors, and male mice display normal anxiety-related behavior [20]. The cKO-T50 mice displayed increased locomotor activity, but the mice finally habituated, suggesting that these mice do not show sustained increases in locomotor activity [20]. BDNF adult knockout mice using an inducible doxycycline system, in which BDNF levels were reduced by ~70% in the hippocampus, were normal in body weight, locomotor activity and levels of aggression [50]. Taken together, it is not inconceivable that the observed elevation of NPC proliferation in the *BDNF cKO *mice used by Chan and colleagues is a reflection of alterations in these neurodevelopmental and/or behavioral parameters. We would argue that in our *BDNF *cKO line, the observed differences in the proliferation of newly-generated hippocampal progenitors in highly-active male WT vs. male cKO mice and reduced survival of these cells reflect *bona fide *effects of reduced postnatal, neuronal expression of BDNF, and not the result of physiological and behavioral processes resulting from near complete loss of postnatal *BDNF*, as reported by Chan et al (2008).

While our Cre lines have the obvious advantage of avoiding developmental consequences of *BDNF *ablation, the activity of the CamKII promoter in the hippocampus is restricted to "differentiated" neurons and therefore we expect that BDNF will still be present, albeit at low levels in the SGL. We have previously demonstrated that SGL cells and neurospheres derived from these cells express BDNF [38]. Moreover, the *CamKII-Cre *allele is not expressed in astrocytes, another source of BDNF. Therefore, it is conceivable that the microenvironment of the SGL in our cKO mice could retain BDNF activity in spite of the overall reduction in the entire hippocampus, and that the cells in the SGL have adequate levels of BDNF necessary for specific physiological responses (e.g. basal proliferation). Thus, it might be also possible that while complete loss of BDNF in the hippocampus affects cell proliferation in adult mice, as observed by Chan et al (2008), approximately 50% reduction in the hippocampus in the postnatal brain does not impair NPC proliferation, but rather, affects NPC survival. In this regard, it is instructive to note that Gao et al (2009) recently reported that mice with conditional deletions of *BDNF *in postnatal-born granular neurons of the DG using a Pro-opiomelanocortin *(POMC)-Cre *allele, in which BDNF levels accumulate to ~50% of wild-type levels, also exhibit impaired survival of newborn cells in standard conditions [51]. These latter studies confirm our findings and support the notion that BDNF plays a significant role in NPC survival.

Notwithstanding the lacunae in our understanding of the cellular and molecular mechanism(s) by which BDNF regulates neurogenesis in the adult DG, future insights in this arena will be essential for developing strategies to isolate and maintain progenitors from adult stem cell niches that may serve as therapeutic modalities for acute and chronic neurodegenerative processes in the adult CNS.

## Methods

### Transgenic mice

Mice that harbor loxP sites flanking the single BDNF coding exon (Exon 5) were previously described [39]. The homozygous "floxed" BDNF mice (BDNF^2lox^/BDNF^2lox^) mice were crossed to mice expressing bacterial Cre recombinase driven by the neuron-specific CaM Kinase II-Cre promoter (CamKII-Cre, line T29-1, [15]). CamKII-Cre/BDNF^2lox^/BDNF^2lox ^and BDNF^2lox^/BDNF^2lox ^mice are referred to as cKO and WT, respectively, in the present study. Generation of conditional BDNF knockout mice using a CamKII-Cre line T50 (cKO-T50) and immunofluorescent labelling for Ki67 were described previously [20,38].

### EE setting and BrdU injections

Animal experiments were conducted in accordance with institutional and NIH guidelines. Cohorts of WT and cKO animals were housed in large cages containing running wheels, tunnels, toys, and chewable materials 3 h a day for 1 month at the age of 3 months. Control groups of animals were maintained in standard laboratory housing conditions. Mice received a single i.p. injection of BrdU (100 mg/kg, Sigma, St. Louis, MO) on the last day of the enrichment. Half of the mice in each group were sacrificed 1 day after the injection to determine progenitor proliferation. The remaining mice were allowed to continue under standard or enriched conditions for 4 weeks and processed to determine survival and neuronal differentiation of the newborn cells. Physical activity levels, defined as the percent time spent on the running wheels, of mice during exposure to EE were observed and recorded every week. Additional mice of each group were processed for ELISA analysis for BDNF protein levels as described below.

### BDNF ELISA

BDNF protein levels were measured using a BDNF ELISA kit (Promega, Madison, WI) according to the manufacture's protocol. Hippocampal or cortical homogenate samples were acidified to pH < 3 with 1N HCl and incubated for 15 min at room temperature, followed by neutralization to pH ~8 with 1N NaOH, as this treatment has been shown to maximize the yield of free protein [52]. Aliquots of 100 μl homogenate samples were incubated in a plate coated with a BDNF antibody and following chromogenic reaction, the resulting absorbance was read in duplicate at 450 nm using ELISA reader (Thermo-Max Microplate Reader and Softmax-Pro software, Molecular Devices, Sunnyvale, CA). Standard curves of pure BDNF were used to quantify the amount of the BDNF in the samples.

### Tissue preparation, and quantify and phenotype of newborn cells

Tissue preparation, and immunofluorescent labelling for BrdU, neuronal nuclei (NeuN), and Glial Fibrillary Acidic Protein (GFAP) were performed as described previously [53]. The antibodies used were rat anti-BrdU (1:100, Accurate Chemical & Scientific Corporation, Westbury, NY), mouse anti-NeuN (1:500, Chemicon, Temecula, CA), and rabbit anti-GFAP (1:500, Dako, Fort Collins, CO). The fluorescent secondary antibodies used were biotinylated donkey anti-rat IgG; Cy2-conjugated Streptavidin; donkey anti-mouse IgG conjugated with Cy5; donkey anti-rabbit IgG conjugated with Cy3 (all 1:250, Jackson ImmunResearch, West Grove, PA).

### Quantitative analyses of morphology and dendritic length of doublecortin (DCX)^+ ^immature neurons

DCX is exclusively expressed in immature neurons and has been widely used as an immature neuronal marker [54]. The brain sections were stained by goat anti-DCX (1:500, Santa Cruz, CA). The immunohistochemical staining was made using the avidin-biotin complex (ABC) system (Vectastain Elite, Vector labs, Burlingame, CA) and nickel-enhanced diaminobenzidine (DAB) incubation. Sections were mounted on gelatin-coated slides, air-dried, counterstained with hematoxylin (vector labs, Burlingame, CA), dehydrated, cleared and coverslipped. 20 DCX^+ ^neurons in each mouse were traced in their entirety and total dendritic lengths were analyzed by NIH Image Software (ImageJ, http://rsb.info.nih.gov/ij). For dendritic complexity, cocentric circle analysis of Sholl (1953) was performed using a separate NIH ImageJ plugin.

### Behavior

Locomotor activity was assessed by placing animals in a new home cage and measuring locomotor activity for 2 h by photobeams linked to computer data acquisition software (San Diego Instruments). Aggressive behavior was assessed using the resident intruder test, as described previously [55], and defined as total number of bites.

### Statistical Analysis

Data are expressed as mean values ± SEM. Error bars in the figures represent SEM.

*T*-test and ANOVA tests were applied to study the relationship between the different variables where appropriate. Values of *p *< 0.05 were considered significant.

## Competing interests

The authors declare that they have no competing interests.

## Authors' contributions

SHC and SSS designed the experiments, statistical analysis, interpreted the results and drafted the manuscript. SHC carried out the majority of the experimental work and YL carried out proliferation study in cKO-T50 mice. SSS and LFP supervised the collection and analysis of all the data. All authors read and approved the final manuscript.
